# Profiles of Perfectionistic Ruminations in Undergraduates: Impact on the Spontaneous Use of Mental Images

**DOI:** 10.3390/ijerph17103488

**Published:** 2020-05-16

**Authors:** María Pilar Aparicio-Flores, José María Esteve-Faubel, Rosa Pilar Esteve-Faubel, Lucía Granados-Alós

**Affiliations:** 1 Department of Developmental Psychology and Teaching, Faculty of Education, University of Alicante, 03690 Alicante, Spain; pilar.aparicio@ua.es; 2 Department of General Didactic and Specific Didactics, Faculty of Education, University of Alicante, 03690 Alicante, Spain; jm.esteve@ua.es (J.M.E.-F.); rosapilar.esteve@ua.es (R.P.E.-F.); 3 Faculty of Education, Valencian International University (VIU), 46002 Valencia, Spain

**Keywords:** mental images, perfectionistic automatic thoughts, future teachers, education, profile

## Abstract

Perfectionistic Automatic Thoughts (PAT) negatively affects people who present it. Hence the importance of their study to determine possible ways of reduction. The current study tried to identify PAT profiles and specify the statistically significant differences in the Spontaneous Use of Mental Imaging in 647 undergraduates. For this the Perfectionism Cognitions Inventory and the Spontaneous Use of Imagery Scale were used. The cluster analyses showed three groups of PATs; low (LPAT), moderate (MPAT) and high (HPAT). An analysis of variance revealed moderate size differences in the visual-spatial capacity as well as in the total of the Spontaneous Use of Mental Images for MPAT and LPAT. Implications for the training of future teachers related to the use of mental imagery that can reduce maladaptive PAT are discussed.

## 1. Introduction

Perfectionism is a complex construct, with a multidimensional approach, which negatively affects people’s daily lives due to the excessive demands that they assume to achieve perfection [1,2]. There are controversies regarding the nature of perfectionism because some authors consider it an adaptive trait whereas others consider it a maladaptive trait [3,4]. However, it is known as a personality trait with a mainly maladaptive nature for the majority of research studies due to its relation with psychopathological variables such as fatigue [2], stress [5], depression [6], neuroticism [7], fear of failure [8] and of humiliation and suicidal ideation [9], among others. Two decades ago, Flett, Hewitt, Blankstein and Gray [10] designed a new inventory (i.e., Perfectionism Cognitions Inventory) to assess the ruminant status of perfectionism, known as Perfectionistic Automatic Thoughts (PAT). PAT are thoughts that provoke the demand of being perfect, pursuing their efforts to achieve this goal and associated to the worries of making any mistakes [11]. Although the fact of being measured as a state, different studies support the temporal stability of PAT [12,13]. These ruminant thoughts are associated to depressive symptoms [14,15,16], psychological distress [17], pessimism [18], neuroticism, stress and negative affect [19], anger [20] and social anxiety [21] among other psychopathologies. 

In spite of the existence of several studies about perfectionism, in recent years less research has been conducted about PAT. Currently, there are significant gaps in the study of PAT profiles, as well as regarding the analyses of differences among these groups in variables of visual character. There are studies that prove that adaptive perfectionism is linked to creativity [22,23]. Notwithstanding, there is lack of analysis regarding perfectionism, or more concretely PAT, and variables such the Spontaneous Use of Imagery (SUI), which is a construct with little research at the moment, especially in the educational sphere.

The SUI, for its part, is known as the ability of using visual mental imagery during their daily thoughts [24]. Its importance is based on the projection of future situations or past memories [25], which can alter the mood of people when these thoughts are linked to negative situations. In this sense, the intrusive mental imagery is associated with an increase of bipolar behaviors, social phobia [26], stress [26,27] and social anxiety [28]. Similarly, the rumination of negative images generates agonizing behaviors in individuals with high levels of perfectionism [29], among others. However, the mental imagery, in positive terms, is also closely related to creativity [30], as well as to self-control, confidence, self-consciousness increase strategies and intrinsic motivation to improve the performance of performed tasks, imagining them before they occur [31,32,33,34]. In fact, at the moment there are studies analyzing the impact on different treatments with positive images on reducing different psychopathologies such as bipolar disorder [35], social phobia [36] and distressing states [26]. Nevertheless, there is still a lack of similar treatments to reduce perfectionism, and more concretely PAT. Consequently, before designing similar treatments to alleviate PAT, the first step that must be contemplated is the strengthening of the link between PAT and SUI, in this last case not only based on negative images. As a result, we find ourselves facing a pioneering work that provides new scientific insight for both variables.

The current study pretends: (a) according to the previous literature observing three groups of PAT in Spanish undergraduate students of early childhood and primary education degrees [21]; and (b) analyzing the differences of SUI between the profiles of PAT.

## 2. Materials and Methods 

### 2.1. Participants

The sample was formed by convenience sampling and it was composed of 647 undergraduates aged between 20 and 36 years old (*M*age = 21.2; *SD* = 5.11), from which 74.81% were female. Of the participants 13.45% affirmed that they were studying and working occasionally. All the participants were studying either the early childhood education degree or the primary education degree during the investigation.

Anonymity of the participants was respected and the undergraduate students that signed the informed consent were the only individuals participating in the study.

### 2.2. Measures

**Perfectionism Cognitions Inventory (PCI)** [10]: the Spanish version of PCI [21] is a self-report measure composed by 17 items and with a 5-point Likert-scale answer (1 = by no means; 5 = all the time), which assesses the PAT frequency from three factors. The first factor “Perfectionistic Efforts”, with 7 items, assesses the thoughts based on a self-oriented sacrifice to achieve perfection (for example, “I can always do better, even if things are almost perfect”). The second factor “Perfectionistic Demands”, with 4 items, assesses perfectionistic reflections and demands of self-improvement (for example, “I should be doing more”). The third factor “Perfectionistic Concerns”, with 6 items, measures those annoying thoughts that differ from performing tasks and standards of performance marked by fear of negative evaluation which can lead to refusal (for example, “Why things cannot be perfect?”). The reliability levels in the study of the Spanish version were acceptable for the total of the scale (α = 0.88) as for the three factors (α = 0.86, 0.71 and 0.83) respectively. 

**Spontaneous Use of Imagery Scale (SUIS)** [37,38]: the Spanish version of SUIS is a self-report scale formed by 9 items and with 5-point Likert-scale answer (1 = quite inappropriate, 5 = entirely appropriate), which assesses the spontaneous use of mental images from three factors [39]. The first factor “Visual-Spatial Capacity”, with 3 items, measures the visual-spatial capacity or intelligence (for example, “When I think in visiting a relative, I almost-always have a mental image of them”). The second factor “Construction of Mental Images by Association”, with 3 items, associates suspected images (for example, “If I see a car which is partially covered behind some bushes, I automatically imagine the car and I visualize it as a whole in my mind”). The third factor “Use of Predictive Images by Experience”, with 3 items, assesses the production of future images due to previously lived experiences (for example, “When I go to dress, I firstly imagine myself with different clothes combinations”). The levels of reliability in the Spanish version were acceptable for the total of the scale (α = 0.75), as well as for their three factors (α = 0.71, 0.72 and 0.73) respectively.

### 2.3. Procedure

After informing the teachers of the interviewed students about the objective of the study, the collaboration of the participants was requested, highlighting the purpose, voluntariness and anonymity. The participants’ informed consent followed the Declaration of Helsinki. 

For administering the questionnaires (PCI and SUIS), a time of approximately 30 min was estimated. Tests were fulfilled by each of the participants during a collective session inside their reference classroom.

### 2.4. Data Analysis

Firstly, a cluster analysis was performed with the non-hierarchical method known as quick cluster analysis. This method was used to establish different profiles of PAT in a sample made by future teachers [40].

Subsequently, an analysis of variance (ANOVA) was performed, with a Bonferroni test for multiple post-hoc comparisons, with the aim of determining the existence of statistically significant differences in SUI scores among the profiles of PAT found. The interpretation of these differences was performed following Cohen’s criteria [39]: >0.80 = large effect size, between 0.50 and 0.79 = medium effect size and between 0.20 and 0.49 = small effect size.

The statistics program SPSS.24 was used for obtaining the results.

## 3. Results

### 3.1. Identification of PAT Groups

**Figure 1:**
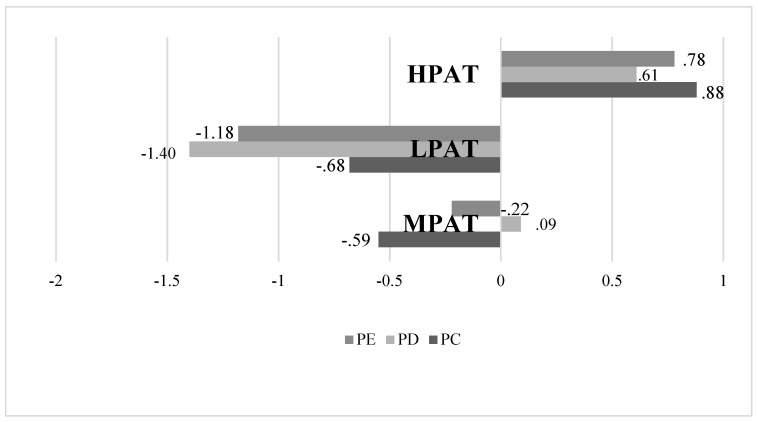
Groups of Perfectionistic Automatic Thoughts (PAT). Note: PC = Perfectionistic concerns; PD = Perfectionistic Demands; PE = Perfectionistic Efforts; MPAT = Moderate Perfectionistic Automatic Thoughts; LPAT = Low Perfectionistic Automatic Thoughts; HPAT = High Perfectionistic Automatic Thoughts.

The cluster analysis revealed three PAT profiles (see Figure 1). A first group with 252 undergraduates (38.95%) and moderate scores in the PCI dimensions. Consequently, this profile was named as Moderate Perfectionistic Automatic Thoughts (MPAT). Another found profile with 131 participants (20.25%) showed low scores in all the PAT factors. As a consequence, this profile was named as the Low Perfectionistic Automatic Thoughts (LPAT). The last group was formed by 264 future teachers (40.80%) and revealed high PAT in the three dimensions of PCI. As a result, this profile was named as the High Perfectionistic Automatic Thoughts (HPAT).

### 3.2. Intergroup Differences

The ANOVA results show the existence of statistically significant differences in the three profiles of PAT in all the SUIS variables. The HPAT profile obtained the highest scores in all the factors and in the total score of the SUIS. Similarly, the MPAT scored higher than LPAT in all the SUIS dimensions (see Table 1). 

Regarding the post-hoc comparisons (Bonferroni test) between PAT groups and SUIS scores, the results show differences of a moderate magnitude (see Table 2) between the HPAT and LPAT groups in visual-spatial capacity, as well as for the total score of the scale, being this last difference of higher magnitude (*p* = <0.001, *d* = 0.59). Moreover, differences of low magnitude are observed between the MPAT and LPAT groups in all the factors and in the total score of the SUIS, with the exception of use of predictive images for experience, as well as between the groups LPAT and HPAT in the construction of mental images by association and use of predictive images for experience. There were no statistically significant differences between the MPAT and HPAT groups.

## 4. Discussion

The current study proposed two objectives: on the one hand, finding different groups of PAT in future teachers aged between 20 and 36 years old and on the other hand, observing the existence of differences in the scores of SUIS between these groups [38].

For the first proposed objective, the observed findings revealed three groups of diverse intensity, composed by low, moderate and high PAT. The MPAT and HPAT were profiles formed by the most part of future teachers, consistent with a previous study that reached similar data [21]. Consequently, it is important to broaden the research in this sense, with the aim of defining specific characteristics of teachers. Additionally, it was observed that perfectionistic demands play a distinct role with regard to perfectionistic concerns and perfectionistic efforts, especially in the MPAT profile. This finding can be explained by the fact that future teachers reflect with PAT to improve their tasks, not linking these reflections to an excessive sacrifice or not presenting strong discomfort while performing tasks [21].

With regard to the second objective, there is a lack of studies that examine the PAT and SUI, as well as perfectionism as a more general trait and SUI. However, it has been proved that higher levels of perfectionism are linked to intrusive mental images more distressing and ruminant [29]. In this sense, it is being investigated that these images influence behavior and the prediction of different levels of psychopathology. Moreover, the studies in which the negativity of these images is transformed by others of positive character are extended, as part of cognitive behavioral treatments that reduce negative thoughts [26,34,35]. However, this type of treatment has not been proven for PAT. Hence there is an importance of testing SUI scores in the population with HPAT and MPAT.

It is important to highlight moderate differences between MPAT and LPAT groups with regard to the visual-spatial capacity, as well as for the total score of SUIS. Moreover, although presenting sizes of low magnitude, differences have been observed between the LPAT group and the HPAT group with regard to construction of mental images by association and use of predictive images for experience. Their link could be indicating that individuals with MPAT and HPAT self-regulate. In other words, the assay of mentally imagining an activity before being performed provides a better performance due to the increase of self-dominance, confidence, self-consciousness and intrinsic motivation [31,32,33,34]. High self-efficacy can trigger higher productivity [33], and individuals with PAT need it to relieve tension. Hence, imagining, before its implementation, different friendship relations or diverse prospective tasks (topics that are evaluated by the SUIS) could give clues about the fact that subjects with MPAT and HPAT present higher SUI as a mental training for improving self-efficacy and reducing tension.

## 5. Limitations and Future Research

It is important to highlight that there were several limitations in the study that must be attended in future works. On one side, the sample was exclusively composed of future teachers, so the results could not be generalized to other undergraduates or the Spanish adult sample. Consequently, future studies should broaden the sample to other adult groups, in order to observe whether the results are characteristic of the teaching staff or can be extended to the whole adult sample. Moreover, considering the novelty of the current work, it is important to examine the obtained results in different age range, cultural and clinical samples, as well as observing differences among sexes.

## 6. Conclusions

To conclude, this study provided a new scientific knowledge, presenting the analyses of different groups of PAT and their differences in SUI scores. The results empirically support a better comprehension of PAT in future teachers, as well as present specific characteristics of this collective that extend the investigation in the fields of education and psychology.

That is to say the results found show diverse educational implications. On the one hand, it is suggested the design of specific treatments that convert PAT, and their possible use of intrusive mental images unrealistic and fanciful, into positive images that develop well-being and reduce psychopathological comorbidities. On the other hand, the fact of discovering among students to be teachers the use of strategies of mental imagery to improve their performance can carry with them a minor use of PAT, improving as well the relation with their students, tolerance and the excessive demand towards them. In this sense, the knowledge about strategies of mental imagery in teachers can lead to the instruction of them between students, which could improve their academic results.

These implications are the primary measures for, subsequently, investigating the design of preventive and reduction treatments for PAT, as well as their maladaptive characteristics [15,16,17,18,19,20,21].

## Figures and Tables

**Table 1:** Differences between PAT profiles and visual mental imagery dimension.

Dimensions	MPAT		LPAT		HPAT		Statistical Significance		
	*M*	*SD*	*M*	*SD*	*M*	*SD*	*F*_(2,644)_	*p*	*η* ^2^
VSC	11.64	2.43	10.38	2.80	11.74	2.52	16.39	<0.001	0.04
CMIA	11.03	2.30	10.40	2.25	11.35	2.21	9.20	<0.001	0.02
UPIE	9.80	2.40	9.32	2.35	10.17	2.45	6.69	0.001	0.02
SUISTotal	32.48	5.16	30.11	5.47	33.27	5.34	18.67	<0.001	0.05

Note: MPAT = Moderate Perfectionistic Automatic Thoughts; LPAT = Low Perfectionistic Automatic Thoughts; HPAT = High Perfectionistic Automatic Thoughts. VSC = Visual-Spatial Capacity; CMIA = Construction of Mental Images by Association; UPIE = Use of Predictive Images for Experience; SUISTotal = Total score of the Spontaneous Use of Imagery Scale.

**Table 2:** Cohen’s d for post-hoc contrast between PAT profiles and dimensions of visual mental imagery.

Dimensions		MPAT and LPAT	MPAT and HPAT	LPAT and HPAT
VSC	*p*	<0.001	n.s.	<0.001
	*d*	0.49	-	0.52
CMIA	*p*	0.02	n.s.	<0.001
	*d*	0.28	-	0.43
UPIE	*p*	n.s.	n.s.	0.002
	*d*	-	-	0.35
SUISTotal	*p*	<0.001	n.s.	<0.001
	*d*	0.45	-	0.59

Note: MPAT = Moderate Perfectionistic Automatic Thoughts; LPAT = Low Perfectionistic Automatic Thoughts; HPAT = High Perfectionistic Automatic Thoughts. VSC = Visual-Spatial Capacity; CMIA = Construction of Mental Images by Association; UPIE = Use of Predictive Images for Experience; SUISTotal = Total score of the Spontaneous Use of Imagery Scale.

## References

[1] Hewitt P.L., Flett G.L. (1991). Perfectionism in the self and social contexts: Conceptualization assessment, and association with psychopathology. J. Pers. Soc. Psychol..

[2] Babapour K.J., Esmaeilpour K., Saeedi S.S. (2015). The role of perfectionism in predicting feeling of cognitive, physical and social fatigue. J. Psychol..

[3] Stoeber J., Otto K. (2006). Positive conceptions of perfectionism: Approaches, evidence, challenges. Pers. Soc. Psychol. Rev..

[4] Muñoz–Villena A.J., Gómez-López M., González-Hernández J. (2020). Perfectionism profiles and anger responses: The relevant role of self-esteem in athletes of professional quarries. Int. J. Environ. Res. Public Health..

[5] Feldman G., Hayes A. (2005). Preparing for problems: A measure of mental anticipatory processes. J. Res. Pers..

[6] Fatemeh G.J., Ghasem N., Majid B. (2012). The relationship between socially prescribed perfectionism and depression: The mediating role of maladaptive cognitive schemas. Procedia Soc. Behav. Sci..

[7] Dunkley D.M., Sanislow C.A., Grilo C.M., McGlashan T.H. (2004). Validity of DAS perfectionism and need for approval in relation to the five-factor model of personality. Pers. Individ. Differ..

[8] Stoeber J., Becker C. (2008). Perfectionism, achievement motives, and attribution of success and failure in female soccer players. Int. J. Psychol..

[9] Pia T., Galynker I., Schuck A., Sinclair C., Ying G., Calati R. (2020). Perfectionism and prospective near-term suicidal thoughts and behaviors: The mediation of fear of humiliation and suicide crisis síndrome. Int. J. Environ. Res. Public Health..

[10] Flett G.L., Hewitt P.L., Blankstein K.R., Gray L. (1998). Psychological distress and the frequency of perfectionistic thinking. J. Pers. Soc. Psychol..

[11] Flett G.L., Hewitt P.L. (2014). The multidimensional assessment of perfectionistic automatic thoughts: A commentary on “examining mutual suppression effects in the assessment of perfectionism cognitions: Evidence supporting multidimensional assessment”. Assess..

[12] Mackinnon S.P., Sherry S.B., Pratt M.W. (2013). The relationship between perfectionism, agency, and communion: A longitudinal mixed methods analysis. J. Res. Pers..

[13] Wimberley T.E., Stasio M.J. (2013). Perfectionistic thoughts, personal standards, and evaluative concerns: Further investigating relationships to psychological distress. Cognit. Ther. Res..

[14] Flett G.L., Hewitt P.L., Demerjian A., Sturman E., Sherry S.B. (2012). Perfectionistic automatic thoughts and psychological of the Perfectionism Cognitions Inventory. J. Ration. Emot. Cogn. Behav. Ther..

[15] Besser A., Flett G.L., Sherry S.B., Hewitt P.L. (2019). Are perfectionistic thoughts an antecedent or a consequence of depressive symptoms? A cross-lagged analysis of the Perfectionism Cognitions Inventory. J. Psychoeduc. Assess..

[16] Casale S., Fioravanti F., Rugai L., Flett G.L., Hewitt P.L. (2019). What lies beyond the superordinate trait perfectionism factors? The perfectionistic self-presentation and perfectionism cognitions inventory versus the big three perfectionism scale in predicting depression and social anxiety. J. Pers. Assess..

[17] Lyubomirsky S., Layous K., Chancellor J., Nelson K.S. (2015). Thinking about rumination: The scholarly contributions and intellectual legacy of Susan Nolen-Hoeksema. Annu. Rev. Clin. Psychol..

[18] Žitniaková-Gurgová B. (2011). Perfectionistic cognitions as related to optimism and pessimism in college students. New Educ. Rev..

[19] Flett G.L., Molnar D.S., Nepon T., Hewitt P.L. (2012). A mediational model of perfectionistic automatic thoughts and psychosomatic symptoms: The roles of negative affect and daily hassles. Pers. Individ. Differ..

[20] Donachie T.C., Hill A.P., Madigan D.J. (2019). Perfectionism and precompetition emotions in youth footballers: A three-wave longitudinal test of the mediating role of perfectionistic cognitions. J. Sport Exercise Psy..

[21] Esteve-Faubel J.M., Aparicio-Flores M.P., Vicent M., Gonzálvez C., Sanmartín R., García-Fernández J.M. (2020). Validation of Spanish version of the Perfectionism Cognitions Inventory: Profiles of automatic perfectionism thoughts and their associations with social anxiety. Prof. Psychol. Res. Pr..

[22] Nekoie-Moghadam M., Beheshtifar M., Mazrae-Sefidi F. (2012). Relationship between employees’ perfectionism and their creativity. Afr. J. Bus. Manage..

[23] Wigert B., Reiter-Palmon R., Kaufman J.C., Silvia P.J. (2012). Perfectionism: The good, the bad, and the creative. J. Res. Pers..

[24] Nelis S., Holmes E.A., Griffith J.W., Raes F. (2014). Mental imagery during daily life: Psychometric evaluation of the Spontaneous Use of Imagery Scale (SUIS). Psychol. Belg..

[25] Andrade J., May J., Deeprose C., Baugh S.J., Ganis G. (2013). Assessing vividness of mental imagery: The Plymouth Sensory Imagery Questionnaire. Br. J. Psychol..

[26] Holmes E.A., Hackman A. (2004). A healthy imagination? Editorial for the special issue of memory. Mental imagery and memory in psychopathology. Mem..

[27] Suh D.E., Chang K.A., Hwang J.U., Kwon J.H. (2019). Prevalence and features of spontaneous recurrent images in social anxiety disorder: Findings from a Korean community sample. Behav. Cogn. Psychother..

[28] Vassilopoulos S.P., Moberly N.J. (2013). Cognitive bias modification in preadolescent children: Inducing and interpretation bias affects self-imagery. Cognit. Ther. Res..

[29] Lee M., Roberts-Collins C., Coughtrey A., Phillips L., Shafran R. (2011). Behavioural expressions, imagery and perfectionism. Behav. Cogn. Psychother..

[30] Daniels-McGhee S., Davis G.A. (1994). The imagery-creativity connection. J. Creat. Behav..

[31] Giacobbi P., Dreisbach K.A., Thurlow N.M., Anand P., García F. (2014). Mental imagery increases self-determined motivation to exercise with university enrolled women: A randomized controlled trial using a peer-based intervention. Psychol. Sport Exerc..

[32] Houghton J.D., Jinkerson D.L. (2007). Constructive thoughts strategies and job satisfaction: A preliminary examination. J. Bus. Psychol..

[33] Sari I. (2015). An investigation of imagery, intrinsic motivation, self-efficacy and performance in athletes. Anthropol..

[34] Spino M.P., Straub W.F. Effect of mental training on the performance of college age distance runners. The Sport Journal.

[35] Holmes E.A., Bonsall M.B., Hales S.A., Mitchell H., Renner F., Blackwell S.E., Watson P., Goodwin G.M., Di Simplicio M. (2016). Applications of time-series analysis to mood fluctuacions in bipolar disorders to promote treatment innovation: A case series. Transl. Psychiat..

[36] Wild J., Hackmann A., Clark D.M. (2007). When the present visits the past: Updating traumatic memories in social phobia. J. Behav. Ther. Exp. Psychol..

[37] Kosslyn S.M., Chabris C.F., Shephard J.M., Thompson W.L. Spontaneous Use of Imagery Scale (SUIS).

[38] Aparicio-Flores M.P., Esteve-Faubel J.M., Vicent M., García-Fernández J.M. Validación española de la Spontaneous Use of Imagery Scale y perfiles del uso espontáneo de imágenes mentales con empatía disposicional [Spanish validation of the Spontaneous Use of Imagery Scale and profiles of spontaneous use of mental images with dispositional empathy].

[39] Cohen J. (1998). Statistical Power Analysis for the Behavioral Sciences.

[40] Hair J.F., Anderson R.E., Tatham R.C., Black W.C. (1998). Multivariate Data Analysis.

